# White Matter Hyperintensities and Functional Outcomes in Patients With Cerebral Hemorrhage: A Systematic Review and Meta-Analysis

**DOI:** 10.3389/fneur.2022.820012

**Published:** 2022-03-21

**Authors:** Qian You, Yi Yang, Hongtao Hu

**Affiliations:** ^1^Department of Neurology, Beijing Jishuitan Hospital, Beijing, China; ^2^Department of Neurology, Beijing Friendship Hospital, Capital Medical University, Beijing, China

**Keywords:** white matter hyperintensities, intracerebral hemorrhage, functional outcome, mortality, meta-analysis

## Abstract

**Background and Objectives:**

There are controversies about white matter hyperintensities (WMH) and the prognosis of spontaneous intracerebral hemorrhage. Our objective is to investigate the relationship between WMH and functional outcomes after intracerebral hemorrhage (ICH).

**Methods:**

We systematically searched PubMed, EMBASE, and Cochrane Library databases from inception through August 10, 2021 without any restriction of countries. Articles investigating the relationship of WMH and functional outcomes as well as mortality of patients with spontaneous ICH were included. We extracted relevant data and evaluated the study quality with the Newcastle-Ottawa Scale. We pooled odds ratio (OR) for the presence and different severities of WMH with random effects models using STATA.

**Results:**

A total of 10,584 patients with ICH in 18 studies were included in the analysis. Moderate/severe WMH were related to poor functional outcome [OR, 1.805, 95% confidence interval (CI), 1.320–2.469] and all-cause mortality (OR, 3.27, 95% CI, 2.07–5.18) after ICH. Besides, the increasing severity of WMH was also related to poor functional outcome (OR, 1.34, 95% CI, 1.17–1.53) and all-cause mortality (OR, 1.62, 95% CI, 1.39–1.90). The pooled data did not find the relationship between the presence of WMH and poor functional outcome (OR, 2.54, 95% CI, 0.91–7.05) after ICH. The results remained stable after adjusting for age, hematoma volume, stroke, and intraventricular hemorrhage.

**Conclusion:**

We found moderate and severe WMH were related to poor functional outcomes and all-cause mortality after ICH. High-quality prospective studies are still needed.

**Systematic Review Registration:**

https://www.crd.york.ac.uk/PROSPERO/, identifier: CRD42021278409.

## Introduction

Cerebral hemorrhage has a high-fatality rate and a high-disability rate. It will be helpful in the judgment of a prognosis if reliable biomarkers can be found. White matter hyperintensities (WMH), one of the imaging manifestations of cerebral small vessel disease, are associated with increased blood brain barrier permeability and loss of microstructural white matter integrity (1), and affect post-stroke remodeling of the myelin sheath (2). Thus, it may influence a post-stroke functional recovery. WMH have been proved to be related to poor functional outcomes after ischemic stroke (3). Several studies have found that WMH are related to poor functional outcomes in patients with spontaneous cerebral hemorrhage, suggesting that WMH are important prognostic factors after intracerebral hemorrhage (ICH) (4). However, the results vary in other studies, probably due to a small sample size or different study methods (5–7). We systematically reviewed and meta-analyzed all published relevant studies to investigate the relationship between WMH and functional outcomes and an all-cause mortality after cerebral hemorrhage.

## Methods

### Protocol and Registration

The study protocol was established before a data extraction process (PROSPERO registration No.: CRD42021278409). We strictly followed the protocol to perform analysis, and we followed the Preferred Reporting Items for Systematic Reviews and Meta-Analyses (PRISMA) guideline to report.

### Search Strategy

We systematically searched PubMed, EMBASE, and Cochrane Library databases from inception through August 10, 2021 without any restriction of language. The search strategy in PubMed is as follows:(((((“Leukoaraiosis”(Mesh)] OR ((((White matter hyperintens^*^(Title/Abstract)] OR [White matter lesion^*^(Title/Abstract)] OR [White matter change^*^(Title/Abstract)] OR [White matter disease^*^(Title/Abstract))] OR [“Cerebral Small Vessel Diseases”(Mesh)] OR [(cerebral small vessel disease^*^(Title/Abstract)] OR [cerebral small-vessel disease^*^(Title/Abstract))] AND [(“Cerebral Hemorrhage”(Mesh)] OR [((((cerebral hemorrhage^*^(Title/Abstract)] OR [intracerebral hemorrhage^*^(Title/Abstract)] OR [cerebral brain hemorrhage^*^(Title/Abstract)] OR [cerebral parenchymal hemorrhage^*^(Title/Abstract)] OR [cerebrum hemorrhage^*^(Title/Abstract)] AND (((outcome) OR (disability)) OR (mortality)). The search strategies for Cochrane and Embase are provided in the Supplementary Materials. We also hand-searched the reference lists of eligible articles and relevant reviews. Two reviewers (QY and YY) performed the search and selected the studies independently, and disagreement was resolved by consulting a third author (HTH).

### Inclusion Criteria

We included all of the eligible cohort studies and case-control studies that investigated the relationship of WMH with functional outcomes or all-cause mortality of ICH. Our primary outcome was functional impairment assessed by modified Rankin Scale (mRS) ≥3 or Glasgow Outcome Scale (GOS) ≤ 3. The secondary outcome was all-cause mortality. Patients with intracranial hemorrhage, including extradural hemorrhage, subdural hemorrhage, and subarachnoid hemorrhage, were excluded. Patients with post-traumatic intracerebral hemorrhage were also excluded. Studies about white matter lesions caused by other than vascular causes, such as inflammatory demyelinating disease and sepsis-associated encephalopathy, were not included. Besides, patients with monogenic causes of stroke, such as cerebral autosomal dominant arteriopathy with subcortical infarcts and leukoencephalopathy, were also excluded. Imaging studies should be performed for assessment of WMH. Studies evaluating WMH by magnetic resonance imaging (MRI) or computed tomography (CT) were both included, as standardized visual rating scales mostly show substantial agreement between CT and MRI (8). In case of an overlap, we included studies with the largest sample size. If the sample size was also the same, the most up-to-date would be included. Besides, we restricted our inclusion to original articles and excluded reviews, case reports, and study protocols.

### Quality Assessment

We evaluated study quality using the Newcastle-Ottawa scale (NOS), ranging from 0 to nine points. Three parts, including selection, comparability, and outcome, were evaluated, respectively, and the total score was recorded.

### Data Extraction

We used a predefined spreadsheet to extract the following information from each article: study characteristics (author, publication year, study design), population-related information [sample size, age, gender, hypertension, GCS on admission, ICH volume, percentage of intraventricular hemorrhage (IVH)], WMH assessment (imaging technique, time to MRI if evaluated by MRI, grading methods of WMH), outcome [definition of outcome, follow-up duration, percentage of poor outcome, odds ratio (OR)/risk ratio (RR)/hazard ratio (HR) and 95% confidence interval (CI)]. If studies presented more than one OR/RR/HR and 95% CI, we extracted the one in the most fully adjusted model. For studies that measured deep WMH (DWMH) and periventricular hyperintensities (PVH) separately and did not provide a global risk estimate for WMH, the results for PVH were used for the meta-analysis. If effect values of more than one time point of follow-up were available in the articles, the longest one would be extracted. QY and YY extracted data independently, and HTH solved the disagreement.

### Statistical Analysis

For functional outcomes and all-cause mortality, pooled analyses including three parts were performed according to the methodology of the articles: (1) comparison between presence and absence of WMH; (2) comparison between moderate/severe and none/mild WMH; (3) ordinal increase in WMH grade and a poor outcome. When studies presented ORs for >1 WMH severity categories, we obtained the effect estimate for moderate/severe vs. mild/none categories using the method suggested by Hamling et al. (9). ORs were presented to evaluate the relationship of WMH and functional outcome as well as mortality. The analyses were performed using the STATA software (V14.0, StataCorp). Random-effects models were used due to several levels of heterogeneity across studies, which were quantified with the I^2^and the Cochran Q statistic. I^2^ exceeding 50 or 75% was considered as moderate and high heterogeneity, respectively.

### Sensitivity Analyses and Subgroup Analyses

Sensitivity analyses were performed to evaluate the stability of results and identify the sources of heterogeneity if they exist. We also performed subgroup analysis according to population information (age), study characteristics (retrospective or prospective, NOS score), imaging of WMH (imaging technique, grading methods of WMH), adjustment factors (age, hematoma volume, symptom severity [NIHSS or Glasgow Coma Scale (GCS)], IVH), follow-up time.

## Results

### Review of Literature

**Figure 1:**
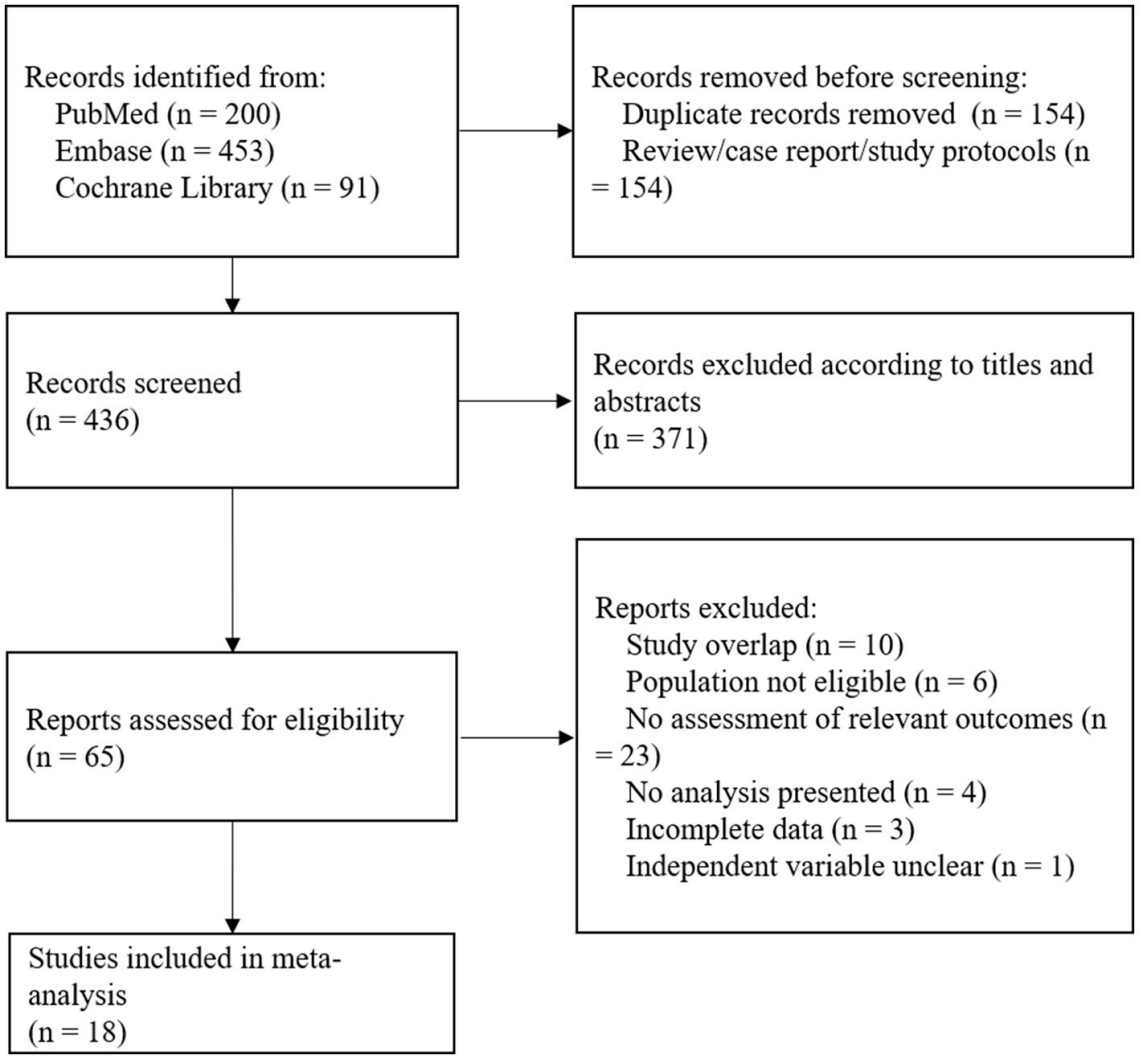
A flow diagram of study selection details. A total of 744 articles were screened for eligibility, and 18 studies were included in the analysis.

Figure 1 illustrated the study selection details. A total of 744 articles were screened for eligibility according to the search strategy described above, including 200 articles from PubMed, 453 articles from EMBASE, and 91 articles from Cochrane. About 590 articles were screened after duplicates were removed, 154 articles were deleted due to review/case/study protocols, and another 371 articles were deleted by titles and abstracts. Following evaluation of full texts, 10 articles were deleted due to an overlap, and eight articles due to data unavailability. About six articles included patients with both ischemic stroke and hemorrhagic stroke without subgroup analyses, and 23 articles did not investigate the effect of WMH on a prognosis of cerebral hemorrhage. Thus, a total of 18 articles were included in the study eventually.

### General Characteristics

A total of 10,584 patients in 18 studies were included in the analysis (Supplementary Table 1), and the median sample size is 304, ranging from 60 to 2,344. Nine studies were prospective, and another nine retrospective. Five studies were derived from randomized controlled trials (6–13). The outcomes of 17 studies were poor prognoses, mostly evaluated by mRS, except one whose outcome was based on GOS. There was only one study whose outcome was functional independence (mRS ≤ 3), and it was listed separately. For functional outcomes, nine studies, six studies, and two studies were included in the comparison of moderate/severe vs. mild/none WMH, a grade increment of WMH, and presence vs. absence of WMH, respectively. For mortality, two articles were included in the moderate/severe vs. mild/none analysis, and two studies were included in the analysis of a grade increment of WMH. About 10 studies used CT to evaluate WMH, and eight studies used MRI. About nine studies adopted van Swieten Scale to score WMH, and nine studies adopted Fazekas scale. Two studies analyzed PVH and DWMH separately instead of as a total score. In such cases, we utilized only the PVH data for meta-analysis (5, 14). In the moderate/severe vs. mild/none analysis, data from the five studies were converted due to multiple WMH severity categories (10, 11, 13, 15, 16).

### Risk of Bias

#### Risk of Bias Within Studies

The Newcastle-Ottawa scale (NOS) was used for evaluation of study quality (Table 1), and the median score was 7, ranging from 4 to 9. There were two articles reaching nine points. Scores of 11 (61.1%) articles were seven points or more. Therefore, the risk of bias in the majority of studies is low. In terms of selection bias, only three studies fulfilled all items, and it was worth mentioning that the evaluation method of WMH in eight studies was MRI, and the representativeness of the exposure group or the case group cannot be scored, because only patients with relatively mild cerebral hemorrhage may be able to perform MRI. Eight studies cannot guarantee that there were no outcome indicators to be observed at the beginning of the study or that the control group cannot guarantee that there was no history of target diseases. In terms of comparability, one study did not adjust for age, two studies did not adjust for symptom severity, two studies did not adjust for hematoma volume, and three studies did not adjust for IVH. In the measurement of an outcome or exposure factors, five studies did not use a blinding method, and six studies did not describe the complete follow-up or non-response rate of exposure factors.

**Table 1:** The NOS score of the included studies.

**Study**	**Selection**	**Comparability**	**Outcome**	**Total**
Caprio et al. (14)	⋆⋆⋆	⋆⋆	⋆⋆	7
Da Silva-Candal et al. (17)	⋆⋆⋆	⋆	⋆⋆⋆	7
Hansen et al. (10)	⋆⋆⋆⋆	⋆⋆	⋆⋆	8
Hostettler et al. (4)	⋆⋆⋆	⋆⋆	⋆⋆⋆	8
Kidwell et al. (18)	⋆⋆	⋆⋆	⋆	5
Kimura et al. (5)	⋆	⋆⋆	⋆⋆⋆	6
Lee et al. (15)	⋆⋆⋆⋆	⋆⋆	⋆⋆⋆	9
Lioutas et al. (19)	⋆⋆	⋆⋆	⋆⋆	6
Morotti et al. (6)	⋆⋆	⋆⋆	⋆⋆	6
Pasi et al. (16)	⋆⋆⋆	⋆	⋆⋆⋆	7
Rodrigues et al. (20)	⋆⋆⋆	⋆⋆	⋆	6
Sato et al. (11)	⋆⋆⋆	⋆⋆	⋆⋆⋆	8
Sykora et al. (12)	⋆⋆⋆	⋆⋆	⋆⋆⋆	8
Tveiten et al. (21)	⋆⋆⋆⋆	⋆⋆	⋆⋆⋆	9
Uniken Venema et al. (13)	⋆⋆	⋆⋆	⋆⋆⋆	7
Warrier et al. (7)	⋆⋆		⋆⋆	4
Won et al. (22)	⋆⋆⋆	⋆⋆	⋆⋆	7
Xu et al. (23)	⋆⋆	⋆⋆	⋆⋆	6

*NOS, Newcastle-Ottawa scale*.

#### Publication Bias

We compiled the 17 articles analyzed above and evaluated the publication bias with Begg's test and Egger's test. The *p*-values were both 0.008, and there was substantial publication bias. We then adopted the method of metatrim. About nine articles were supplemented, and the change of effect values between before and after metatrim was small, indicating that, although there is a certain publication bias, the original results are robust.

### Synthesis of Results

#### Primary Outcome

**Figure 2:**
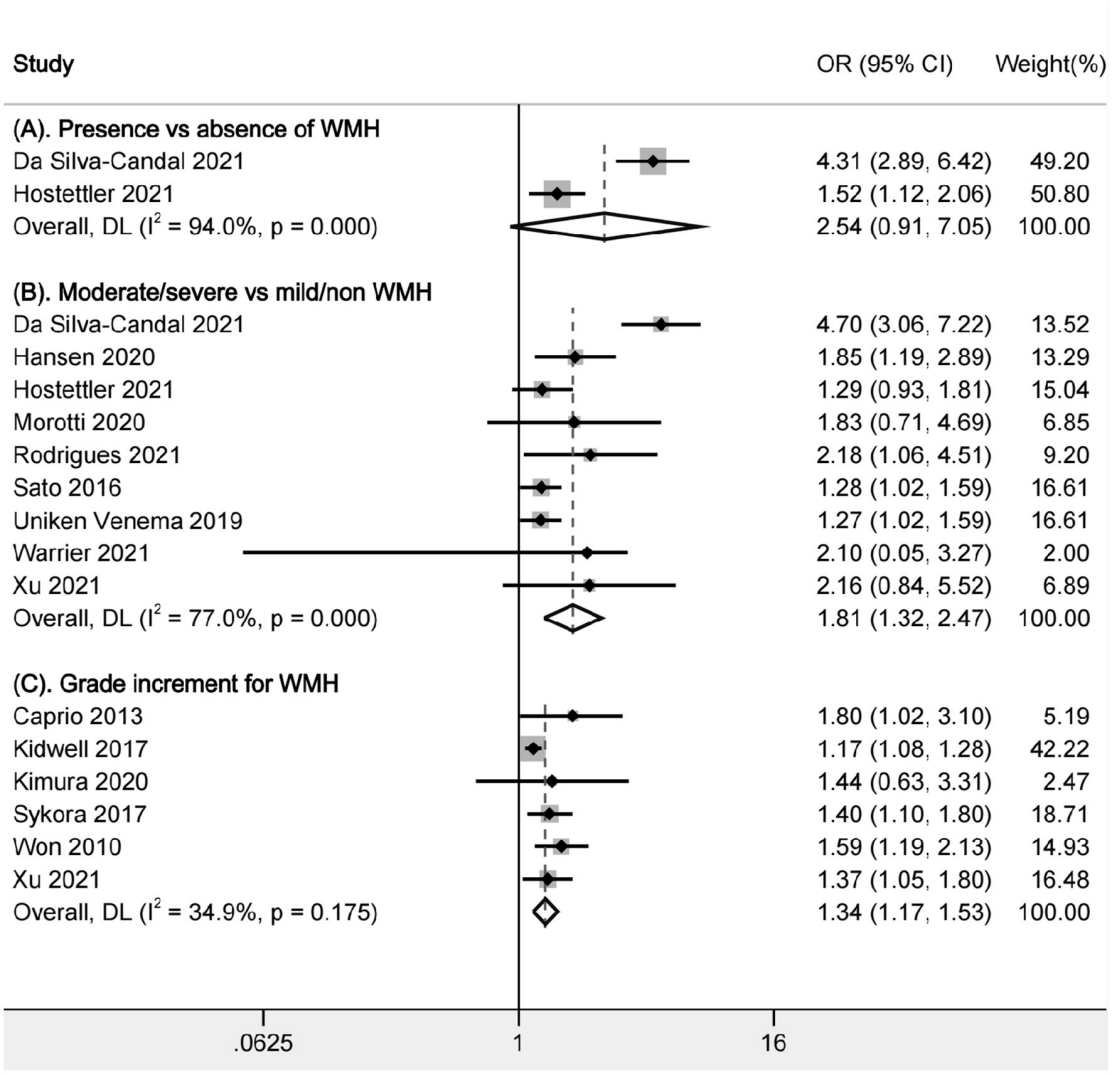
Pooled analysis of relationship between WMH and poor functional outcome. **(A)** Comparison between presence and absence of WMH; **(B)** Comparison of functional outcomes between moderate/severe and none/mild WMH; **(C)** Ordinal increase in a WMH grade. WMH: white matter hyperintensities.

In the comparison of presence vs. absence of WMH, only two studies were available (Figure 2A), and the heterogeneity was substantially high (I^2^ = 94.0%). The pooled data did not find the relationship between the presence of WMH and poor functional outcome (OR, 2.54, 95% CI, 0.91–7.05), although the two relevant articles showed positive results (OR, 4.31, 95% CI, 2.89–6.42; OR, 1.52, 95% CI, 1.12–2.06). There were differences between the study design (prospective for one and retrospective for another), grading methods of WMH (Fazekas scale for one, and van Swieten scale for another) and adjustment factors.

In the comparison of moderate/severe vs. mild/none WMH, a total of 7,375 patients in 9 articles were included (Figure 2B). Moderate/severe WMH were related to poor functional outcomes after ICH (OR, 1.805, 95% CI, 1.320–2.469, I^2^ = 77%). There was high heterogeneity; thus, sensitivity analysis was performed to explore the reason. We found that the heterogeneity was mainly related to one article Da Silva-Candal et al. (17) (Table 2). In the study investigating the relationship between the severity of WMH and good functional outcomes after ICH, no association was found between the severity of WMH and functional independence (OR, 0.61, 95% CI, 0.2–1.93) (19).

**Table 2:** Sensitivity analysis of WMH and functional outcomes.

**Study**	**OR**	**95%CI**		**I^**2**^**	***P* (Cochrane's Q)**
		**Lower limit**	**Upper limit**		
**Moderate/severe vs. mild/non-WMH**					
Da Silva-Candal et al. (17)	1.37	1.20	1.56	0.0%	0.576
Hansen et al. (10)	1.81	1.27	2.57	79.4%	0.000
Hostettler et al. (4)	1.93	1.34	2.79	79.3%	0.000
Morotti et al. (6)	1.81	1.30	2.52	79.8%	0.000
Rodrigues et al. (20)	1.77	1.27	2.48	79.3%	0.000
Sato et al. (11)	1.95	1.32	2.88	77.8%	0.000
Uniken Venema et al. (13)	1.95	1.32	2.88	77.6%	0.000
Warrier et al. (7)	1.80	1.31	2.48	79.8%	0.000
Xu et al. (23)	1.78	1.28	2.48	79.6%	0.000
**Grade increment for WMH**					
Caprio et al. (14)	1.31	1.15	1.49	32.70%	0.203
Kidwell et al. (18)	1.46	1.26	1.70	0.0%	0.881
Kimura et al. (5)	1.35	1.16	1.56	47.10%	0.109
Sykora et al. (12)	1.34	1.14	1.59	40.30%	0.153
Won et al. (22)	1.26	1.13	1.41	15.60%	0.315
Xu et al. (23)	1.35	1.15	1.60	43.80%	0.130

*WMH, white matter hyperintensities*.

In the analysis of a grade increment for WMH, a total of 1,492 patients in 6 articles were included in the analysis (Figure 2C). Increasing severity of WMH were related to poor functional outcomes after ICH (OR, 1.34, 95% CI, 1.17–1.53, I^2^ = 34.90%). There was low heterogeneity across studies. Besides, we also performed sensitivity analysis and found that the results were stable after excluding any of the articles.

#### Secondary Outcome

**Figure 3:**
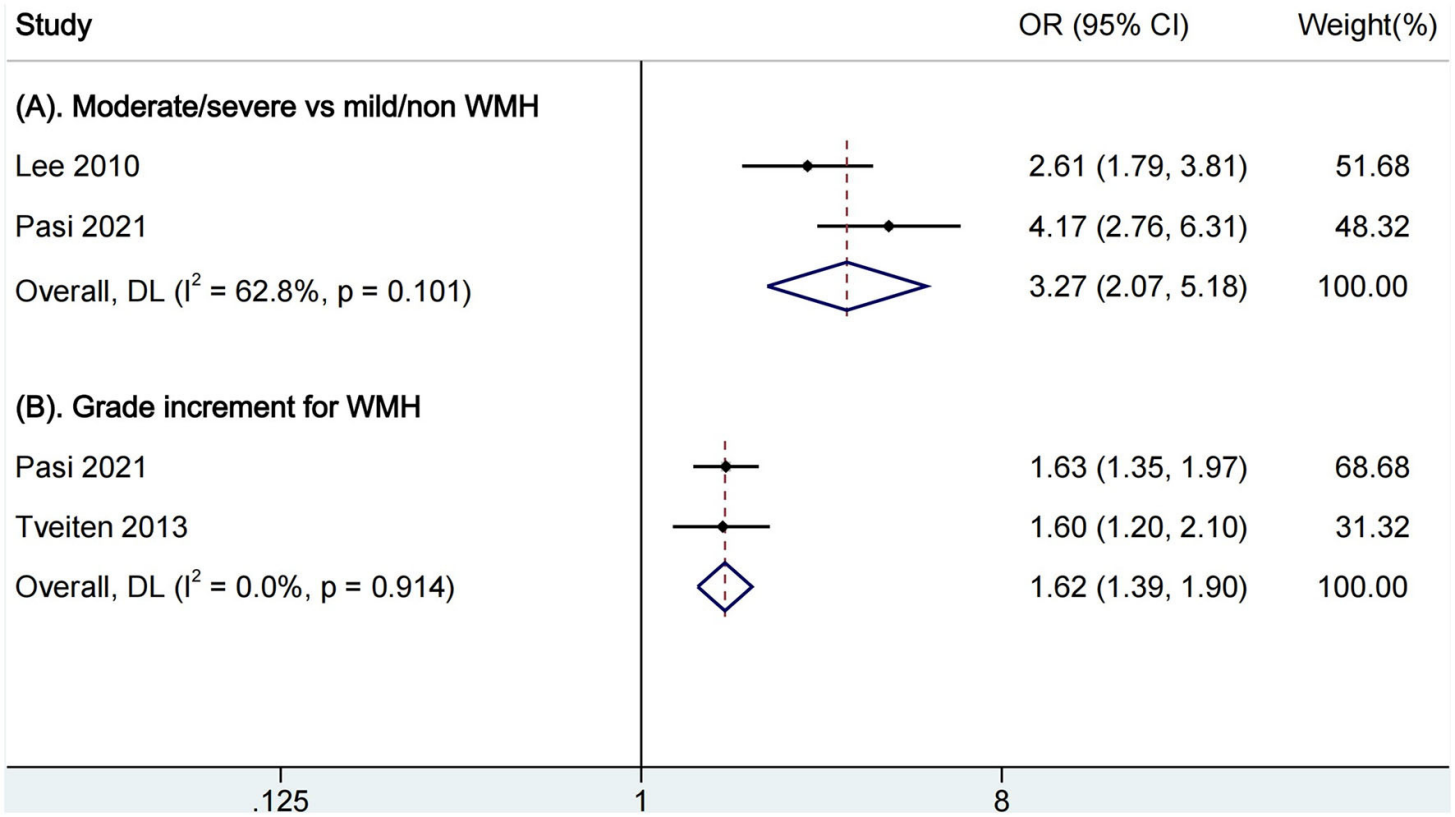
Pooled analysis of relationship between WMH and all-cause mortality. **(A)** Comparison of functional outcomes between moderate/severe and none/mild WMH; **(B)** Ordinal increase in a WMH grade. WMH, white matter hyperintensities.

In the comparison of moderate/severe vs. mild/none WMH, two articles were included for pooled analysis of mortality (Figure 3A), and moderate/severe WMH were related to increased mortality (OR, 3.27, 95% CI, 2.07–5.18, I^2^ = 62.8%). The heterogeneity is moderate. In the analysis of a grade increment for WMH, two studies were available for analysis (Figure 3B). We found increasing WMH were related to increased mortality (OR, 1.62, 95% CI, 1.39–1.90, I^2^ = 0.0%). There was not an obvious heterogeneity. There were no studies available for comparison of presence vs. absence of WMH.

#### Subgroup Analyses

The results remained stable in all of the subgroup analyses whether: age exceeded 65 years old, the study was retrospective or prospective, the study quality was high or low, WMH were evaluated by CT or MRI, by Fazekas scale or Van Swieten scale, or the results were adjusted by age, hematoma volume, symptom severity (NIHSS or GCS) or IVH (Table 3). Besides, we found that moderate/severe WMH were related to a poor outcome after ICH whether short term or long term.

**Table 3:** Subgroup analysis of WMH and functional outcomes.

**Variables**	**N**	**I^**2**^**	**OR**	**95%CI**		***P* (Heterogeneity)**
				**Lower limit**	**Upper limit**	
**Moderate/severe vs. mild/non WMH**						
Age (years)						0.230
≥65	3	90.8	2.36	0.97	5.74	
<65	6	0.0	1.36	1.18	1.57	
Study						0.557
Retrospective	4	90.5	1.89	1.09	3.27	
Prospective	5	0.0	1.58	1.24	2.01	
Imaging						0.756
CT	6	85.3	1.78	1.24	2.55	
MRI	3	0.0	2.00	1.06	3.77	
Grading method						0.007
Fazekas scale	4	38.2	3.00	1.71	5.26	
Van Swieten Scale	5	4.8	1.35	1.17	1.55	
NOS score						0.570
≥7	5	87.9	1.74	1.17	2.57	
<7	4	0.0	2.07	1.29	3.34	
**Adjusted factors**						
Age	8	79.8	1.80	1.31	2.48	0.887
Hematoma volume	7	82.4	1.78	1.27	2.50	0.690
Severity	7	0.0	1.37	1.20	1.56	0.000
IVH	6	0.0	1.42	1.21	1.67	0.382
Follow up						0.528
<1year	7	81.9	1.75	1.23	2.49	
≥1year	2	0.0	2.17	1.22	3.85	
Value						0.981
OR	8	79.8	1.81	1.30	2.52	
RR	1	/	1.83	0.71	4.70	
**Grade increment for WMH**						
Age (years)						0.825
≥65	2	0.0	1.40	1.11	1.78	
<65	4	54.2	1.36	1.13	1.63	
Study						0.255
Retrospective	3	0.0	1.47	1.23	1.77	
Prospective	3	38.5	1.27	1.07	1.52	
Imaging						0.125
CT	2	0.0	1.48	1.22	1.78	
MRI	4	12.6	1.24	1.09	1.40	
Grading method						0.125
Fazekas scale	4	12.6	1.24	1.09	1.40	
Van Swieten Scale	2	0.0	1.48	1.22	1.78	
NOS score						0.020
≥7	3	0.0	1.51	1.26	1.80	
<7	3	0.0	1.19	1.10	1.30	
**Adjusted factors**						
Age	6	34.9	1.34	1.17	1.53	0.175
Hematoma volume	5	43.8	1.35	1.15	1.60	0.130
Severity	6	34.9	1.34	1.17	1.53	0.175
IVH	5	47.1	1.35	1.16	1.56	0.109
Follow up						0.933
<1year	5	43.8	1.35	1.15	1.60	
≥1year	1	/	1.37	1.05	1.80	

*IVH, intraventricular hemorrhage; WMH, white matter hyperintensities; MRI, magnetic resonance imaging; CT, computed tomography; NOS, Newcastle-Ottawa Scale; OR, odds ratio; RR, risk ratio*.

## Discussion

A total of 10,584 patients with ICH in 18 studies were included in the study. We compared the presence or absence of WMH and mild to severe WMH, respectively, and found that presence of WMH may not be related to the poor functional outcome of cerebral hemorrhage, but moderate and severe WMH were associated with functional disability. The results were stable in the subgroups that included patients of different ages, used CT or MRI, adopted different WMH scoring scales, and adjusted for age, symptom severity, hematoma volume, and IVH. In addition, moderate and severe WMH were related to an all-cause mortality of intracerebral hemorrhage.

As for the functional outcome, we did not find a relationship between the presence of WMH and a poor outcome of ICH, but the small number of articles and substantial high heterogeneity made the result doubtful. More high-quality studies are still needed. Moderate/severe WMH were found to be associated with poor functional outcomes after ICH. Considering the high heterogeneity, we performed sensitivity analysis, which revealed that one article contributed to heterogeneity. We studied this article carefully and found that it was the only one evaluating WMH by Fazekas scale on CT. The results were not adjusted by symptom severity or IVH but adjusted by a soluble tumor necrosis factor-like weak inducer of apoptosis (sTWEAK) whose interaction with ICH growth was associated with poor functional outcomes. Different grades of WMH classified by Fazekas scale were all found to be related to the poor functional outcome in the article. The analysis of a grade increment for WMH also suggested that the increasing grade of WMH was associated with poor functional outcome. However, the study investigating the good functional outcome after ICH has found no clear correlation between functional independence and the severity of WMH (19). More studies are still needed.

As for the mortality outcome, moderate and severe WMH were found to be associated with all-cause mortality after intracerebral hemorrhage, and an increasing grade of WMH was also shown to be related. However, the sample sizes were both small. There were no articles available to study whether the presence of WMH affects the mortality of cerebral hemorrhage. Thus, more studies are still needed.

An underlying mechanism of the impact of WMH on functional outcome after ICH remains poorly understood. It has been shown that an increased blood brain barrier permeability and loss of microstructural white matter integrity are related to an unfavorable post-stroke outcome, while WMH burden is a predictor of diffuse blood brain barrier permeability (1). Studies have found that there are often changes in the composition of the extracellular matrix of cerebral blood vessels in cerebral small vessel diseases, which further affect the differentiation of oligodendrocyte precursor cells into oligodendrocytes and the formation of new myelin segments. Therefore, WMH may be related to the abnormal function of oligodendrocyte precursor cells and remodeling of the myelin sheath, thereby affecting functional recovery (2). In addition, WMH may be associated with hematoma growth, which indicates poor functional outcome after ICH (7, 17). Moreover, the patients with severe WMH usually have vascular risk factors, such as older age and hypertension, so increased risk of cardio-cerebral vascular events may explain higher mortality.

There were limitations in our study. First, deaths were not excluded in the primary outcome, which was defined by a poor functional prognosis. Second, the number of included studies was limited, especially in the part of comparison between presence and absence of WMH. Only two articles were included for analysis, and the heterogeneity is high. However, the results of the other two parts were basically the same and remained stable in the sensitivity analyses and subgroup analyses. Thus, we concluded that moderate-to-severe WMH were related to the poor functional prognosis of cerebral hemorrhage, while the association of the functional outcome with mild WMH remained undetermined. In addition, there is a relative lack of studies on mortality. More relevant studies are still needed. Last but not least, effect values provided in the articles on the functional outcomes were mostly OR and a few presented as RR. We pooled OR and RR for analysis. In this case, we performed subgroup analyses according to OR or RR, and found that the result was stable.

Our study has several strengths. The study was carried out based on a pre-defined protocol and registered on PROSPERO. We pooled the data of 10,584 patients from 18 studies and performed the analyses according to different WMH severity classifications from original articles. Our research is rigorous and up-to-date so as to investigate the relationship between the severity of WMH and the functional prognosis of cerebral hemorrhage.

## Conclusion

We found moderate and severe WMH were related to poor functional outcomes and all-cause mortality in patients with ICH. High-quality prospective studies are still needed.

## Data Availability Statement

The original contributions presented in the study are included in the article/Supplementary Material, further inquiries can be directed to the corresponding author.

## Author Contributions

QY proposed the idea, evaluated the publication bias, and drew the first manuscript. QY and HH were in charge of the study design. QY and YY performed the search, selected studies, extracted data independently, and made statistical analysis, and HH solved the disagreement. YY interpreted data. The article was carefully checked and revised by HH. All authors read and approved the final manuscript.

## Conflict of Interest

The authors declare that the research was conducted in the absence of any commercial or financial relationships that could be construed as a potential conflict of interest.

## Publisher's Note

All claims expressed in this article are solely those of the authors and do not necessarily represent those of their affiliated organizations, or those of the publisher, the editors and the reviewers. Any product that may be evaluated in this article, or claim that may be made by its manufacturer, is not guaranteed or endorsed by the publisher.
