# Supplementary material for: White Matter Hyperintensities and Functional Outcomes in Patients With Cerebral Hemorrhage: A Systematic Review and Meta-Analysis
Source: Front Neurol. 2022 Mar 21;13:820012. doi: 10.3389/fneur.2022.820012 (PMC8978301; doi:10.3389/fneur.2022.820012)
Supplement: Supplementary file 1 [file Table_1.DOCX]

**Supplemental Table 1 Characteristics of the included studies**

| **Author**  **(year)** | **Study design** | **Sample size**  **(n)** | **Age** | **Male**  **(%)** | **Hypertension**  **(%)** | **GCS score on admission** | **Hematoma volume**  **(ml)** | **IVH**  **(%)** | **Imaging for WMH** | **Time to imaging if MRI** | **Assessment of WMH** | **Definition of outcome** | **follow up** | **poor outcome**  **(%)** |
| --- | --- | --- | --- | --- | --- | --- | --- | --- | --- | --- | --- | --- | --- | --- |
| Fan Z. Caprio et al.  (2013) | Prospective | 95 | 63.9±13.5 | 49 | 79 | 14 (12–15) | 8.75 (2.8-18.7) | NA | MRI | median 1.8(95%  CI, 0.9-4) days | Fazekas scale | mRS 4-5 | 28days and 3 months | NA |
| Andrés da Silva-Candal et al.  (2021) | Retrospective | 653 | 71.1±11.9 | 56 | 70.60 | NA | 40.35±36.82 | 26.45^a^ | CT | / | Fazekas scale | mRS ≥3 | 3months | 53.14 |
| Björn M. Hansen et al.  (2020) | Prospective | 635 | 59(51-67) | 58.11 | 91 | 10 (7–13) | 10.2  (4.4-20.5) | 87 | CT | / | van Swieten Scale | mRS ≥4 | 6months | 55.28 |
| Isabel C. Hostettler et al.  (2021) | Prospective | 864 | 73.6±11.9 | 58.10 | 67.10 | 13.9±1.9 | 14.9±20.2 | 29.60 | CT | / | van Swieten Scale | mRS ≥3 | 6months | 61.92 |
| Chelsea S. Kidwell et al.  (2017) | Prospective | 600 | 60.8±13.6 | 55.50 | 79.60 | 13.3±3.0 | 9.1  (3.5-20.8) | 34.40 | MRI | 1.6  (0.8-3.4)days | modified Fazekas score | mRS ≥4 | 3months | 36.00 |
| Yoko Kimura et al.  (2020) | Retrospective | 144 | 65.0±12.2 | 67.40 | 88.90 | NA | 18.5±19.1 | 37.50 | MRI | 11.8±13.2days | Fazekas scale | mRS≥3 | discharge | 68.06 |
| S.-H. Lee  et al. (2010) | Prospective | 1321 | 60.32±13.08 | 54.90 | 59.20 | Mild  (>12.5) : 52.5%  Moderate (8.5–12.4) :27.8%Severe  (<8.4) : 19.7% | 10.44  (4.20-25.88) | 29.90 | CT | / | van Swieten Scale | mortality | 30days;  long-term :32.62±16.07months | 9.3(30days); 29.8(long-term) |
| Vasileios-Arsenios Lioutas et al.  (2018) | Retrospective | 111 | 72(61-82) | 56.76 | 72.07 | 14 (11-15) | 20.7  (6.4-34.9) | 42.34 | MRI | Median of 2 days | Fazekas scale | mRS≤3 | 3months | 36.04 |
| Andrea Morotti et al.  (2020) | Retrospective | 195 | 61.64±12.94 | 60.50 | 75.90 | Normal  (15) : 61.54%  Mild  (12-14) :28.72%  Severe  (3-11) : 9.7% | NA | 19.79 | MRI | NA | Fazekas scale | mRS≥3 | 3months | NA |
| Marco Pasi et al.  (2021) | Prospective | 304 | 67.6±14.0 | 54.90 | 65.50 | NA | NA | NA | MRI | NA | Fazekas scale 0-3 | mortality | 10(8-10.5)years | 57.80 |
| Mark A Rodrigues et al.  (2021)^21^ | Prospective | 402 | 78(68-84) | 46 | 65.92 | Alive (44.28%)  14(14-15)  Death (55.72%)  11(6-14) | 20(6-55) | 47 | CT | / | van Swieten Scale | mRS ≥4 | 1year | 73.3 |
| Shoichiro Sato et al.  (2016) | Retrospective | 2069 | 63.74±13.02 | 62.64 | 72.06 | NA | Group1(53.46%)  13.8(7.5-24.5)  Group2(46.54%)  8.2(3.9-13.7) | 28.85 | CT | / | van Swieten Scale | mRS ≥3 | 3months | 53.46 |
| Marek Sykora et al.  (2017) | Retrospective | 262 | 67.3±14 | NA | NA | 14 (range 6-15, IQR 4) | 18.1(31.5) | 28.60 | CT | / | van Swieten Scale | mRS ≥3 | 3months | 66.1 |
| Arnstein Tveiten et al.  (2013) | Prospective | 134 | 75.3±12.0 | 55.22 | 53.73 | Mild  (13-15) : 61.19%  Moderate  (5-12) : 21.64%  Severe  (3-4) : 16.42% | 17.5(5-44) | 37.31 | CT | / | van Swieten Scale | mortality | 30days;  long-term:4.7(2.5-6.6)years | 37(30 days);  62.69(long-term) |
| Simone M. Uniken Venema et al.  (2019) | Retrospective | 2344 | 61.33±14.31 | 59.56 | 80.50 | Group1 (24.87%)  15（15-15）  Group2 (75.13%)  14（11-15） | Group1 (24.87%)  4.32  (1.50-10.1)  Group2 (75.13%)  12.19 (4.95-27.1) | 36.99 | CT | / | van Swieten Scale | mRS 3-5 | discharge | 75.1 |
| Anand R. Warrier et al.  (2021) | Prospective | 60 | 54.08±11.57 | 78 | 75 | NA | 31.47±21.18 | 38.33 | MRI | 54.53±46.48hrs | Fazekas's scale | mRS ≥4 | 3months | 47.46 |
| Yu Sam Won et al.  (2010) | Retrospective | 238 | 59.85±12.78 | 60.50 | 55.46 | Group1 (55.88%)  15(12-15)  Group2 (44.12%)  12(8-14) | 26.01±28.96 | 32.35 | CT | / | van Swieten Scale | GOS 1–3 | 3months | 44.10 |
| Mangmang Xu et al.  (2021) | Prospective | 153 | 61.40±12.27 | 72.55 | 80.39 | Group1 (69.93%)  13.7±2.3  Group2 (30.07%)  12.2±3.0 | Group1 (69.93%)  9.4(3.0-19.7)  Group2 (30.07%)  10.0(3.7-24.3) | 23.53 | MRI | 6.4  (3.9-15.8)days | Fazekas scale | mRS ≥4 | 4.9(3.1-6.0)years | 30.1 |

^a^percentage of IVH or subarachnoid hemorrhage

GCS:Glasgow Coma Scale; IVH:intraventricular hemorrhage; WMH:white matter hyperintensities; MRI:magnetic resonance imaging; CT:computed tomography; mRS:modified Rankin Scale.
